# Supplementary figures and images for: TCA cycle remodeling drives proinflammatory signaling in humans with pulmonary tuberculosis
Source: PLoS Pathog. 2021 Sep 24;17(9):e1009941. doi: 10.1371/journal.ppat.1009941 (PMC8494353; doi:10.1371/journal.ppat.1009941)

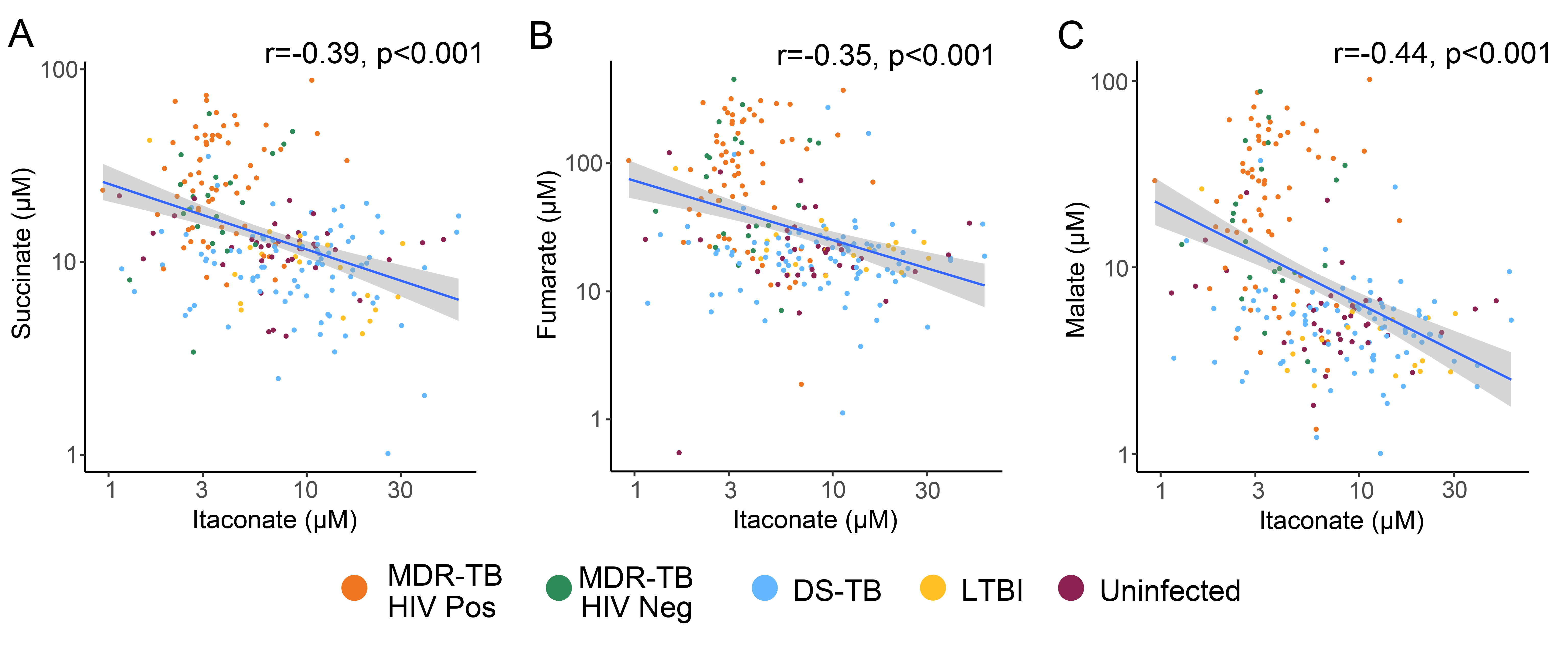

Supplement: S1 Fig — Plasma concentrations of itaconate were significantly and negatively correlated with TCA cycle intermediates (A) succinate, (B) fumarate and (C) malate. (TIF) [file ppat.1009941.s001.tif]

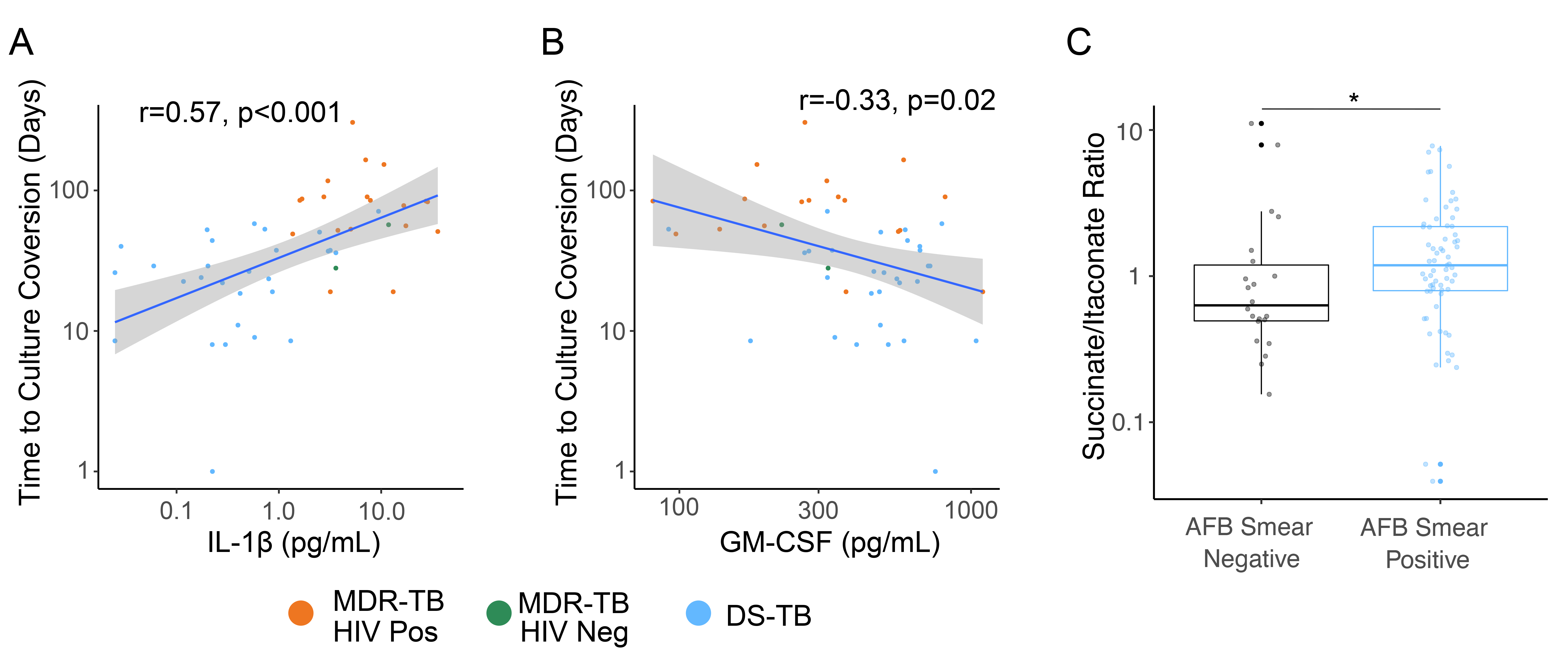

Supplement: S2 Fig — (A) Time to culture conversion was significantly and positively correlated with plasma concentrations of IL-1β at study enrollment and (B) negatively correlated with concentrations of GM-CSF. (C) In persons with drug susceptible (DS)-TB, the plasma ratio of succinate to itaconate was significantly increased in those with a persistently positive sputum smear for acid-fast bacilli (AFB) at enrollment versus those who converted to a negative AFB sputum smear. (TIF) [file ppat.1009941.s002.tif]

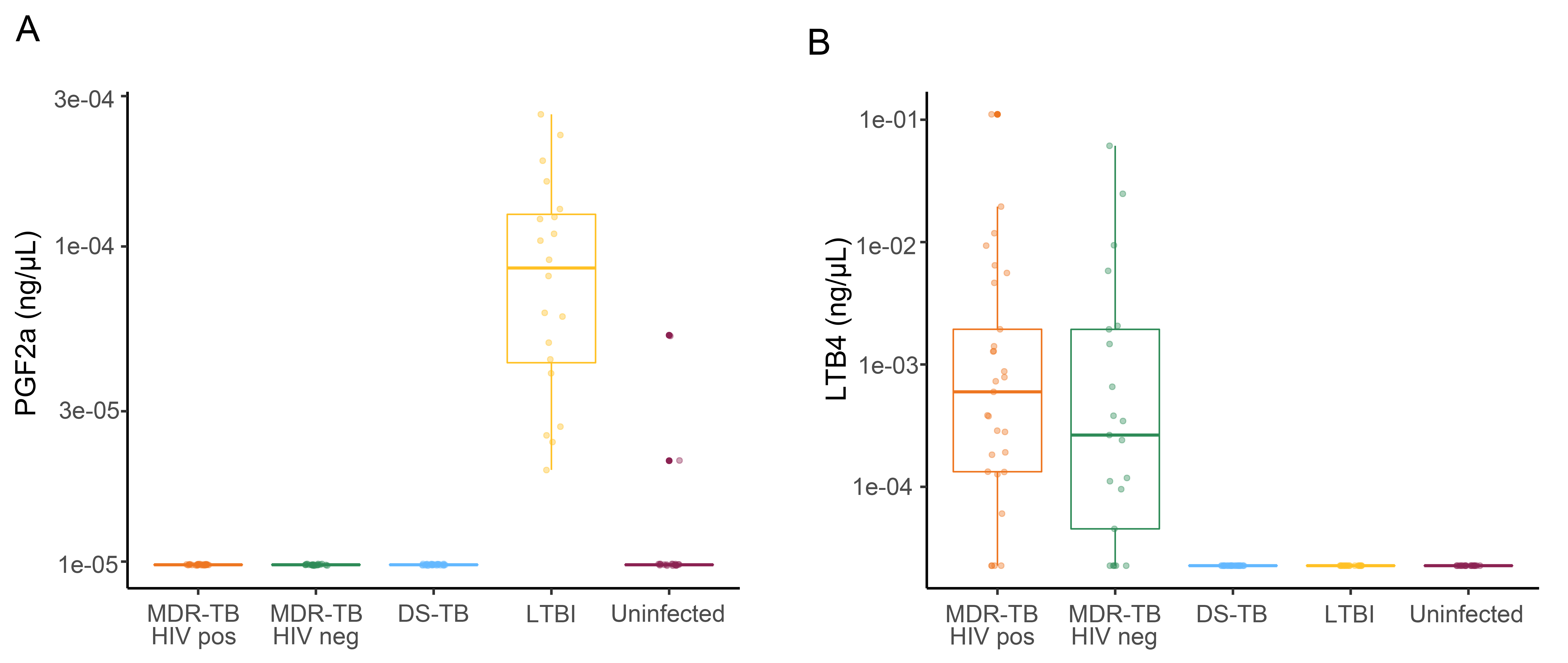

Supplement: S3 Fig — (A) Plasma concentrations of PGF2a were below the limit of detection in all TB disease groups as well as most uninfected U.S. controls while all persons with LTBI had detectable plasma concentrations. (B) LTB4 was only detected in plasma in persons with MDR-TB. (TIF) [file ppat.1009941.s003.tif]

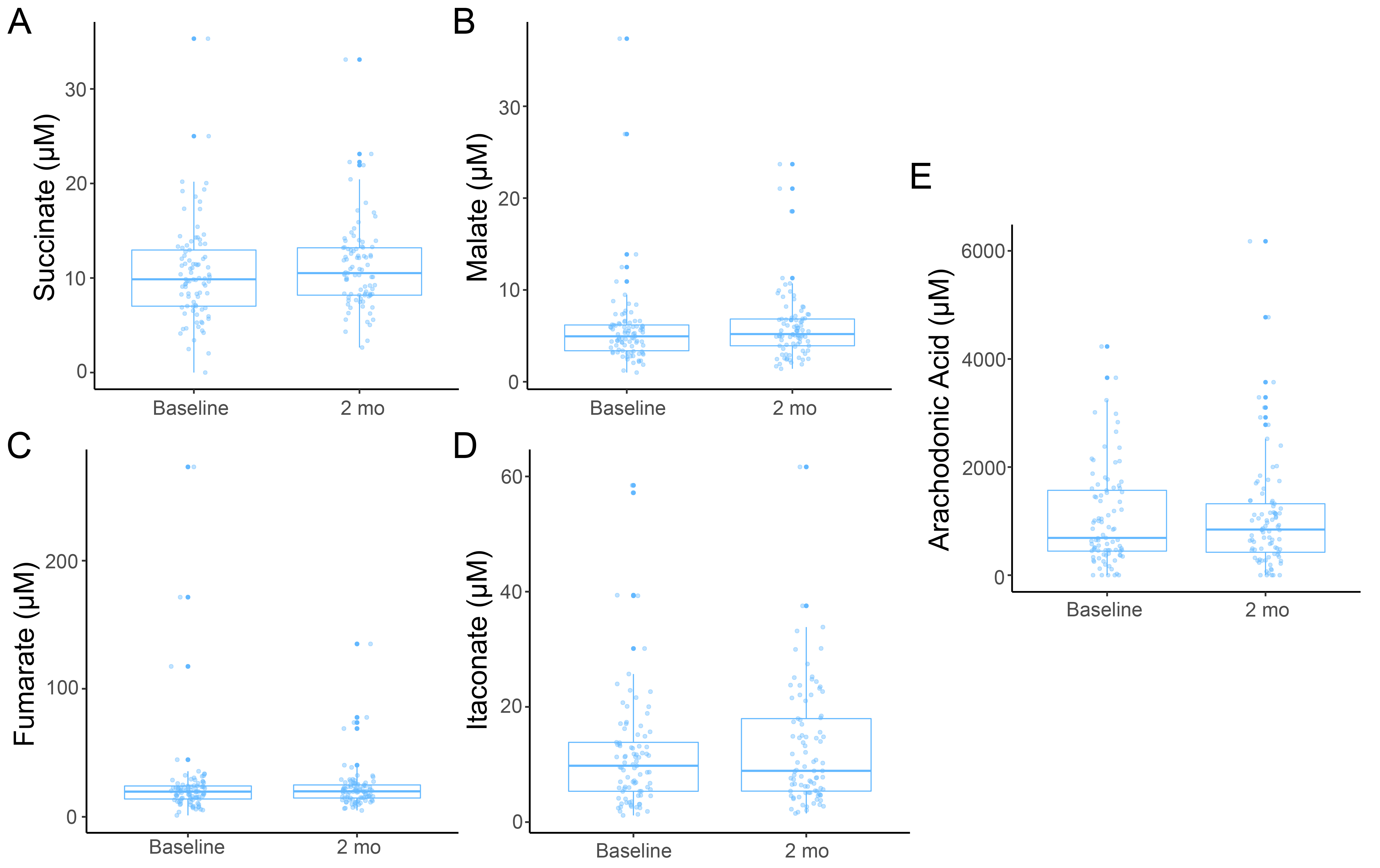

Supplement: S4 Fig — Plasma concentrations of (A) succinate, (B) Malate, (C) fumarate, (D) itaconate and (E) arachidonic acid were not significantly different after 2 months of treatment versus baseline in persons with drug-susceptible TB from Georgia (n = 89). (TIF) [file ppat.1009941.s004.tif]

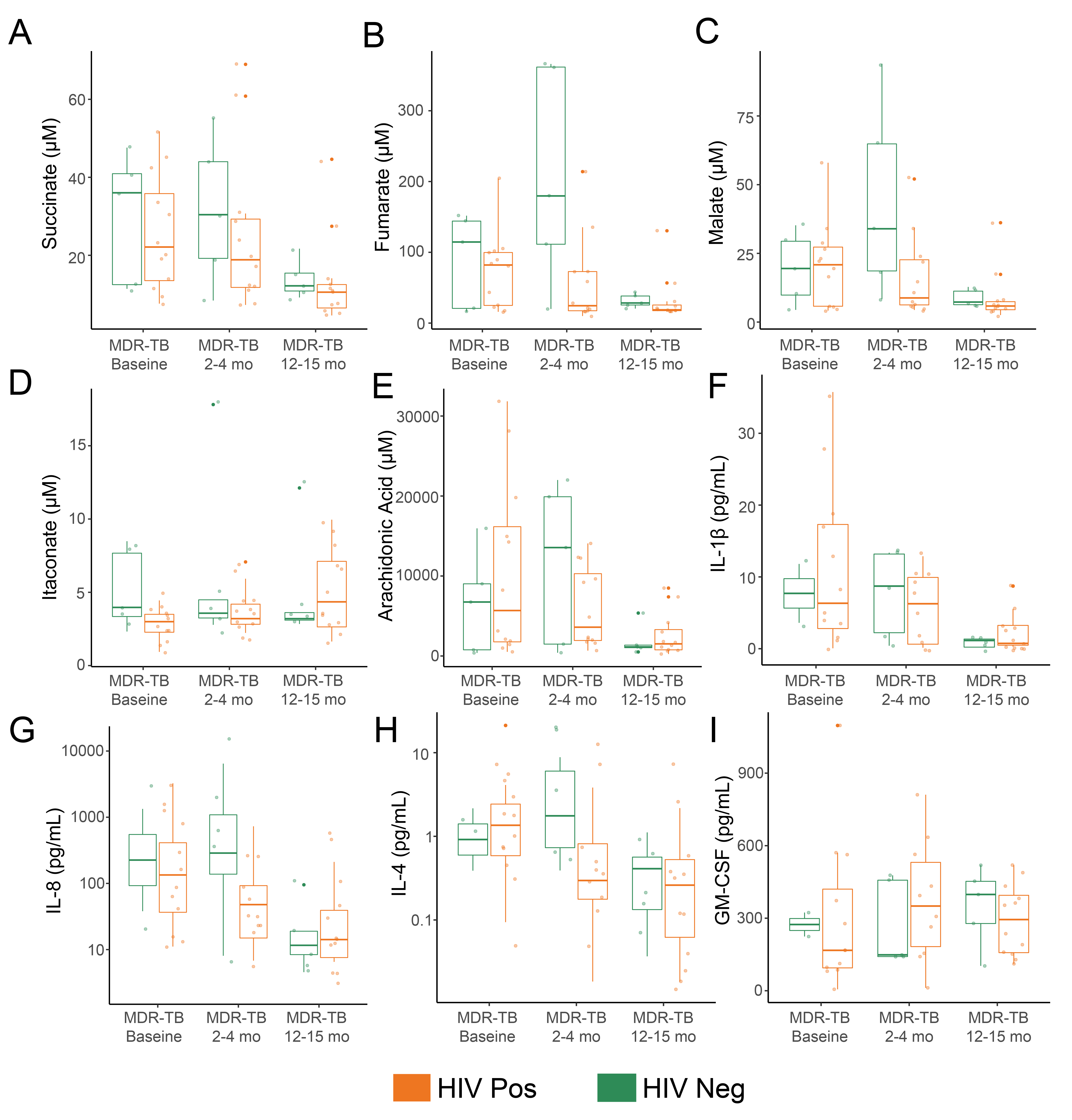

Supplement: S5 Fig — Decreases in proinflammatory metabolites and cytokines with MDR-TB treatment was similar in persons with MDR-TB with (orange; n = 12) and without (green; n = 5) HIV co-infection. In both groups, plasma concentrations of itaconate and GM-CSF were unchanged over time. (TIF) [file ppat.1009941.s005.tif]
