# Supplementary material for: TCA cycle remodeling drives proinflammatory signaling in humans with pulmonary tuberculosis
Source: PLoS Pathog. 2021 Sep 24;17(9):e1009941. doi: 10.1371/journal.ppat.1009941 (PMC8494353; doi:10.1371/journal.ppat.1009941)
Supplement: S1 Table — (DOCX) [file ppat.1009941.s006.docx]

| **Participant Characteristics** | **MDR-TB HIV positive (n=31)** | **MDR-TB HIV negative (n=6)** | **DS-TB (n=29)** | **Controls without Mtb infection (n=20)** |
| --- | --- | --- | --- | --- |
| Female sex, n (%) | 18 (58) | 11 (52) | 14 (48) | 15 (75) |
| Age, years (median [IQR]) | 35 (28-41) | 40 (39-52 | 32 (23-38) | 44 (38-55) |
| CD4, cells/mm^3^ (median [IQR]) | 229 (161-373) | N/A | N/A | N/A |
| HIV Viral Load, copies/mL (median [IQR]) | 136 (<40-2,062) | N/A | N/A | N/A |
| TB disease history, n (%)  No TB history  Yes, completed treatment  Yes, failed treatment | 5 (16)  16 (52)  10 (32) | 0 (0)  2 (33)  4 (67) | 29 (100) | N/A |
| +AFB smear at first study visit, n (%)* | 13 (59) | 3 (50) | 23 (79) | N/A |
| +Sputum culture at diagnosis, n (%) | 64 (100) | 21 (100) | 30 (100) | N/A |
| +Sputum culture at first study visit, n (%)** | 21 (88) | 3 (50) | 25 (86) | N/A |
| Time to sputum culture conversion, days (median [IQR]) § | 84 (53-90) | 43 (35-50) | 26 (11-38) | N/A |

Multidrug resistant (MDR); Drug susceptible (DS)

*AFB sputum smear results were not available for 9 MDR-TB participants (24%)

**AFB sputum culture results were not available for 7 MDR-TB participants (19%)

§Data on sputum culture conversion was missing for 3 MDR-TB participants (13%) with a positive sputum culture at baseline
