# Supplementary material for: TCA cycle remodeling drives proinflammatory signaling in humans with pulmonary tuberculosis
Source: PLoS Pathog. 2021 Sep 24;17(9):e1009941. doi: 10.1371/journal.ppat.1009941 (PMC8494353; doi:10.1371/journal.ppat.1009941)
Supplement: S2 Table — (DOCX) [file ppat.1009941.s007.docx]

| **Participant Characteristics** | **MDR-TB HIV positive (n=29)** | **MDR-TB HIV negative (n=21)** | **DS-TB (n=30)** | **LTBI (n=20)** | **Controls without Mtb infection (n=19)** |
| --- | --- | --- | --- | --- | --- |
| Female sex, n (%) | 16 (55) | 11 (52) | 15 (50) | 9 (45) | 14 (74) |
| Age, years (median [IQR]) | 35 (26-40) | 28 (20-48) | 32 (23-37) | 39 (28-44) | 47 (37-55) |
| CD4, cells/mm^3^ (median [IQR]) | 218 (132-350) | N/A | N/A | N/A | N/A |
| HIV Viral Load, copies/mL (median [IQR] | 162 (<40-40,400) | N/A | N/A | N/A | N/A |
| TB disease history, n (%)  No TB history  Yes, completed treatment  Yes, failed treatment | 7 (24)  13 (45)  9 (31) | 9 (43)  6 (29)  6 (29) | 30 (100) | N/A | N/A |
| +AFB smear at first study visit, n (%)* | 9 (45) | 8 (50) | 24 (80) | N/A | N/A |
| +Sputum culture at diagnosis, n (%) | 29 (100) | 21 (100) | 30 (100) | N/A | N/A |
| +Sputum culture at first study visit, n (%)** | 19 (86) | 16 (80) | 26 (87) | N/A | N/A |
| Time to sputum culture conversion, days (median [IQR]) § | 85 (54-90) | 57 (53-80) | 25 (10-38) | N/A | N/A |

Multidrug resistant (MDR); Drug susceptible (DS)

*AFB sputum smear results were not available for 14 MDR-TB participants (28%)

**AFB sputum culture results were not available for 8 MDR-TB participants (16%)

§Data on sputum culture conversion was missing for 7 MDR-TB participants (20%) with a positive sputum culture at baseline
