# Supplementary material for: TCA cycle remodeling drives proinflammatory signaling in humans with pulmonary tuberculosis
Source: PLoS Pathog. 2021 Sep 24;17(9):e1009941. doi: 10.1371/journal.ppat.1009941 (PMC8494353; doi:10.1371/journal.ppat.1009941)
Supplement: Attachment — Submitted filename: Response to Reviewers.docx [file ppat.1009941.s014.docx]

**RESPONSE TO REVIEWERS**

**Reviewer #1**

**Reviewer comment:** The current study highlights important differences between MDR TB-cohort and DS-TB cohorts/controls. However, the high prevalence of HIV co-infection limits the enthusiasm of the reviewer. Given the known effect of viral infection on host metabolism and eicosanoid production, the co-infection with HIV could lead to under- or over-estimation of the response. The authors should consider separating the MDR-TB cohort into HIV+ and HIV- throughout the study.

**Author response:** The reviewer correctly points out that HIV infection can have profound effects on host metabolism and inflammatory signaling. As requested by the reviewer, we separated the HIV positive and HIV negative participants in the MDR-TB group throughout the revised manuscript. This demonstrates that differences in metabolic and inflammatory signaling seen in the MDR-TB group did not differ significantly between HIV positive and HIV negative participants for any of the parameters measured.

**Reviewer comment:** The control groups were not clear as it appears to be a mix of LTBI to healthy individuals. I would suggest the authors to separate the LTBI from Healthy controls for the analysis.

**Author response:** The LTBI group was separated from the uninfected group throughout the revised manuscript as suggested by the reviewer. The MDR-TB group demonstrated significantly increased plasma concentrations of TCA cycle metabolites and proinflammatory eicosanoids versus those with and without LTBI, as well as those with drug-susceptible TB.

**Reviewer comment:** In Figure 3, the data shows that several cytokines are correlated with TCA cycle intermediates/succinate. Why did the authors choose to focus on IL1-beta, considering the existent literature that MDR limits IL1beta production by inducing IFN-I (Howard et al., 2018, Nat Microbiol)?

**Author response:** We focused our analysis on IL-1β because it had the strongest correlation with plasma concentrations of TCA cycle intermediates succinate, fumarate and malate, and succinate has previously been shown to drive IL-1β production in activated macrophages (PMID: 23535595). Further, the finding that IL-1β is increased in MDR-TB is supported by independent human studies (PMID: 26445573). Small clinical trials of inhaled IFN-alpha have shown benefit in treatment-refractory MDR-TB (PMID: 10094322), possibly by reducing secretion of IL-1β (PMID: 9769275).

The reviewer correctly points out that in their 2018 Nature Microbiology paper, Howard et al. found that IL-1 production was reduced in macrophages infected with *Mtb* strains containing specific *rpoB* mutations and that *Il1r* -/- mice infected with these drug-resistant *Mtb* strains did not experience the same increase in *Mtb* burden as seen in *Il1r* -/- mice infected with drug-susceptible strains of *Mtb*. While this study demonstrated that resistance mutations in *Mtb* genes that produce phenotypic drug resistance can alter immunometabolic host responses, it is important to note that these findings were based on *in vitro* and mouse models and that this altered host response was not observed in all *rpoB* mutants. Additionally, a reduced role for IL-1β signaling during the initial stages of *Mtb* infection does not preclude a more important role in driving pathologic inflammation during later stages of TB disease progression. To our knowledge, differences in immunometabolic responses in MDR-TB have yet to be described in humans. In the present study, we found systemic metabolic changes fueling persistent inflammation in later stages of human TB disease that would be difficult to capture using *in vitro* or mouse models. We have added further discussion on this important point to the Discussion of the revised manuscript (page 17-19, lines 273-314).

**Reviewer comment:** GM-CSF levels are negatively correlated with succinate. Given the predominant role of GM-CSF in combination with antibiotic treatment (Benmerzoug et al., 2018, Scientific Reports), the authors should perform the same experiments as in Figure 3B-D with IL1-beta.

**Author response:** The reviewer correctly points out that GM-CSF plays an important role in regulating pathologic inflammation in TB disease (PMID: 29066547, PMID: 33149141). In the present study, GM-CSF was significantly decreased in the MDR-TB group and plasma concentrations were negatively correlated with concentrations of TCA cycle intermediates succinate, fumarate, and malate as well as proinflammatory eicosanoids. Thus, we agree with the reviewer that low concentrations of GM-CSF representant an important part of the inflammatory immunometabolic network seen in the MDR-TB group. The additional analyses suggested by the reviewer have been added to the revised Figure 3 and Figure 5. We also included a more detailed discussion of the GM-CSF results in the revised manuscript (pages 11-12, lines 138-155 and page 19, lines 315-332).

**Reviewer comment:** In Figure 5, please show a heatmap or correlation between the different oxylipins and TCA metabolites as in Figure 3. In addition, the authors demonstrate that infection with MDR is associated with a shift towards increased production of lipoxygenase derived eicosanoids, such as 5-HETE and 12-HETE. What is the effect of MDR on prostanoids (e.g. PGE2) or lipoxins (e.g. lipoxin A4) which have been previously linked to susceptibility/resistance to TB (PMID: 20622882, PMID: 18955568, PMID: 24990750 ). Is there any correlation between these particular eicosanoids and GM-CSF?

**Author response:** As suggested by the reviewer, we revised Figure 5 to show a heatmap of correlations between plasma concentrations of proinflammatory eicosanoids and TCA cycle metabolites and cytokines. Similar to TCA cycle intermediates, plasma concentrations of GM-CSF were significantly and negatively correlated with proinflammatory eicosanoids.

PGE2 and lipoxin A4 were not measured as part of the eicosanoid panel in the present study. Prostaglandin F2-alpha was measured but was not detected in plasma in all TB disease cohorts. Leukotriene B4 was also measured and was increased in both HIV-positive and HIV-negative persons with MDR-TB. These findings are consistent with upregulation of proinflammatory eicosanoid signaling in the MDR-TB group. These results were added as Supplemental Figure 3 to the revised manuscript.

**Reviewer comment:** In general, is there any correlation between the metabolic and lipidomic reprogramming observed with MDR-TB and circulating levels of immune cells in comparison to DS-TB or controls? What is the level of protection from the macrophages derived from MDR-TB individuals compared to DS-TB?

**Author response:** We agree with the reviewer that understanding that distribution and transcriptional profiles of immune cell subsets associated with the metabolic phenotypes observed in the present study is important. However, we were unable to conduct these experiments due to limited of availability of peripheral blood mononuclear cells. We hope to build on these findings in future mechanistic studies.

Reviewer comment: On Line 792 Immonometabolic should be immunometabolic

**Author response:** This change was made in the revised manuscript.

Review In Figure 6, Please show the controls on the same graphs demonstrating the return to baseline following TB treatment.

**Author response:** We added the control group to the longitudinal treatment data from the MDR-TB group displayed in Figure 6 of the revised manuscript.

**Reviewer #2**

**Reviewer comment:** This study has been performed on a multi-cohort group of patients who were recruited for other studies. The cohort of MDR-TB patients were recruited from a previous study designed to assess treatment outcomes for HIV+/MDR patients vs HIV-/MDR patients. The cohort for the study under review has a 75% positivity rate for HIV infection – we are not given any information on the CD4 counts, viral load titres, treatment regimen (if any), treatment duration etc. These patients were recruited before the introduction of WHO recommendations to treat people diagnosed with HIV at diagnosis and would therefore likely have been subject to previous guidelines where HIV treatment was based on CD4 counts. These patients have likely therefore had a period of unsuppressed HIV infection, before having a CD4 count low enough to merit therapy. Considering there are studies looking at the effects of HIV on cellular metabolism, this is a highly relevant confounding factor on their study selection. The authors sate that the reported changes are not due to HIV. Other important features of this study group (by review of reference 13) are that the HIV positive cohort are 70% female and over 70% have been previously treated for TB, compared to 46% female and 46% previously treated for TB in the HIV- group. It’s unclear how these baseline characteristics carried over to the selected cohort for the current study.

**Author response:** We agree with the reviewer that uncontrolled HIV, low CD4 counts, and receipt of ART are known confounders of systemic cytokine and metabolite profiles (PMID: 34237254). However, as noted above, we found no significant differences in plasma concentrations of TCA cycle metabolites, cytokines or proinflammatory eicosanoids in HIV-positive versus HIV-negative persons with MDR-TB. This strongly suggests observed differences in immunometabolic remodeling between groups were driven by TB disease rather than HIV co-infection. To demonstrate that the MDR-TB cohort did not differ based on HIV status, we sepaeated the MDR-TB group into those with and without HIV co-infection throughout the revised manuscript. We added information about prior TB treatment in the revised manuscript tables, as well as information about CD4 count, viral load, and receipt of ART in the HIV-positive group as suggested by the reviewer.

**Reviewer comment:** In relation to the DS and control groups: both of these groups differ in country of origin to the MDR group. A study in PNAS looked at differences in the crystal structure of the itaconic acid pathway enzymes (https://doi.org/10.1073/pnas.1908770116) and found major differences between people of different ethnicity, specifically a higher rate of a mutation related to enzyme activity among African ethnicity compared to American, Asian and European. Comparing differences in this immune pathway across groups of different ethnicity is therefore challenging.

**Author response:** The reviewer correctly points out that the groups in the present study were enrolled from different geographic locations. In the study of *Irg1* polymorphisms (which encodes cis-aconitate decarboxylase [CAD]) cited by the reviewer, it should be noted that the CAD polymorphism present in nearly 20% of those with African ancestry was associated with increased CAD activity and would therefore be expected to result in greater production of itaconate. Further, it is unknown whether the polymorphisms described in this study result in altered plasma concentrations of itaconate. Among control participants in the present study (5/20 with LTBI and 10/37 uninfected controls had African ancestry) we did not find any association between African ancestry and plasma concentrations of itaconate (Wilcoxon rank sum test p=0.74). However, given the impact of polymorphisms in *Irg1* on plasma concentrations of itaconate in humans is unknown, we have added this as a limitation to the study in the revised discussion (page 20, lines 337-341).

**Reviewer comment:** Rates of concurrent (infectious) illness, or of previous exposure is not discussed, and most likely varies between the groups – considering the significant differences in disease epidemiology between South Africa and the United States.

**Response:** Rates of concurrent infectious illnesses such as helminth infections were not measured in these populations. Given the metabolic remodeling seen in the MDR-TB group was reversed with effective treatment for MDR-TB, this suggests the observed metabolic phenotypes were due to complications of TB disease rather than unmeasured co-infections. We have added this as a study limitation in the revised manuscript (page 20, lines 337-341).

**Reviewer comment:** The Control group seems to consist of people with LTBI and those without – why are these not separated? This makes it hard to disentangle differences between those with LTBI, those with exposure and no LTBI, and those without prior exposure.

**Response:** The reviewer correctly points out that those with and without LTBI were combined into a single control group in the original analysis. This was done because the two groups had minimal differences in plasma concentrations of TCA cycle metabolites. We separated these groups throughout the revised manuscript as suggested by the reviewer.

**Reviewer comment:** The authors have examined plasma samples taken from patients over the length of a treatment course for MDR/DS-TB. Each of these cohorts will be on differing drug regimens, with different administration frequencies, with potentially individual effects on cellular metabolism. This extends further to the HIV+ cohort, who, based on the original cohort study information, are mostly also on ART during the study period. Any changes in glycolytic/oxidative metabolism caused by drug therapy might lead to changes in TCA cycling, and therefore metabolite production/signaling. Furthermore, sampling the MDR-TB and DS-TB groups at 12-15 months presumably means one groups has finished drug therapy and the other not. The control group is also not on any drug therapy, at any point. This has not been acknowledged.

**Author response:** The treatment response data shown in Figure 6 is for the MDR-TB cohort alone. Plasma samples for the DS-TB cohort were analyzed after 2 months of treatment and there was no difference in any of the measured TCA cycle metabolites compared to baseline in this group. This data is now shown in Supplemental Figure 4. The absence of change in TCA cycle metabolites in the DS-TB group over time makes it unlikely the observed metabolic changes in the MDR-TB group were due to receipt of antibiotics. As stated in the manuscript, plasma samples over the course of treatment were only available for a subset of the MDR-TB cohort (n=17). This included 5 HIV-negative and 12 HIV positive individuals. We also added a figure showing the trend in plasma concentrations of cytokines and metabolites over time in those with MDR-TB stratified by HIV status (Supplemental Figure 5). This figure shows that all metabolites and cytokines declined with adequate MDR-TB treatment regardless of HIV status, indicating these metabolic changes were not due to receipt of ART or improved control of HIV viremia.

**Reviewer comment:** The other issue with sample analysis is to draw conclusions about disease in the pulmonary compartment by studying plasma samples. Bronchoalveolar lavage samples have not been studied. As the authors state themselves, the microbiome contributes significantly to succinate presence in the plasma. This study seemed to be limited by samples that had been previously collected during the original MDR-TB treatment study (reference 13).

**Author response:** The reviewer correctly points out that BAL fluid samples were not available from these study participants. While it is possible that some signaling pathways localized to the lung may be missed by not studying BAL, analysis of plasma samples has the advantage of capturing systemic differences in immunometabolic remodeling. This limitation has been added to the revised Discussion (Page 20, lines 341-344).

**Reviewer comment:** Assessment of lipidomics is performed on a “subset” of the original population – we are given no information, however, of the rate of HIV infection or other baseline characteristics in this group. This is also the case for the cytokine analysis (Fig 6).

**Author response:** The reviewer correctly points out that the lipidomics and cytokine assays were performed on a subset of the overall population. In the MDR-TB group, we attempted to include as many HIV-negative participates as sample availability would allow. We included a table of the participant characteristics in each subset in the supplemental material of the revised manuscript (Supplemental Table 1 and Supplemental Table 2).

**Reviewer comment:** The study is limited by lack of mechanistic analysis. It is acknowledged by the authors that the results are observational, however they draw a bold conclusion that the IL-1B/eicosanoid signaling pathway could be targeted by HDT: I would feel it is difficult to jump to this conclusion with observational data, particularly in a pathway this novel. The relationship between itaconate and Mtb in humans is somewhat unexplored in even in vitro studies – one would imagine mechanistic interrogation would be quite important to understand given the various roles of itaconate that we currently know about (inhibition of SDH, stabilization of NRF2, inhibition of isocitrate lyase).

**Author response:** The reviewer correctly points out that the role of itaconate and other TCA cycle intermediates in regulating inflammation in TB disease is not yet fully understood. Thus, we agree more mechanistic studies are in order before suggesting that cell-permeative forms of these metabolites could be used as host-directed therapies. We have therefore revised the discussion section to include a discussion about the potential roles for itaconate in TB disease, which, as the reviewer points out, remain relatively unexplored in *in vitro* and animal models (page 19, lines 315-332).

**Reviewer #3**

**Reviewer comment:** One shortcoming of this paper is the lack of follow up studies to test the mechanisms and the model presented. While this is understandable given the human patients, it does raise questions that are important to consider. For example, the authors cannot distinguish whether the increased inflammation creates an environment that drives bacterial replication or if uncontrolled bacterial replication is driving inflammation. This is not entirely addressable by the nice inclusion of drug treatment conditions but could be addressed more explicitly in the discussion.

**Author response:** The reviewer correctly points out that in this observational study we are unable to determine whether metabolic remodeling is a function of uncontrolled bacterial growth, or whether the host response itself drives differences in bacterial replication and survival. We explicitly highlight the importance of further elucidating the causal pathway of this observation in the Discussion section of the revised manuscript (page 18, line 296-299).

**Reviewer comment:** Do the authors think the metabolic reprogramming is solely driven by the host response, or could factors encoded by Mtb also contribute to this process. For example, the ESX-1 secretion system is known to disrupt mitochondria would this accelerate the switch to glycolysis?

**Author response:** While we believe propagation of host inflammatory cascades due to ineffective treatment is the primary cause of immunometabolic alterations in the MDR-TB group in the present study, we agree with the reviewer that alterations in *Mtb* itself could also contribute to immunometabolic remodeling. As noted above, certain mutations in *rpoB* have been shown to produce altered immunometabolic phenotypes in *in vitro* and mouse models of *Mtb* infection. As the reviewer points out, the ESX-1 secretion system in different *Mtb* lineages has also been shown to produce differential effects on the mitochondria of host cells (PMID: 31879349, PMID: 27500737), which has the potential to rewire host metabolic responses in different ways. Thus, it will be important in future studies to determine the relative contribution of factors encoded in *Mtb* on the metabolic phenotypes produced in humans with TB disease. We have expanded our discussion of this possibility in the revised manuscript (pages 18-19, lines 300-314).

**Reviewer comment:** Did the authors examine IL1Ra which was shown in the mouse model to also drive disease and over-ride elevated IL1b and shutting down IL1R signaling?

**Author response:** We would like to thank the reviewer for pointing out the need to assess other IL-1β family cytokines. In ongoing ex vivo human and non-human primate model studies (primarily focused on chronic HIV/SIV infection) by our group, we have quantified plasma levels of IL-1β, IL-1a, IL-1Ra, IL-18 and IL-33. The data from these studies indicates that plasma concentrations of IL-1β are significantly correlated with IL-1a levels (Spearman's correlation test; p<0.05), while plasma levels of IL-1Ra are negatively correlated with plasma IL-1a levels (Spearman's correlation test; p<0.05). The correlations that were observed between IL-1β, IL-1a and IL-1Ra in these cohorts, did not hold true for IL-18 and IL-33. Given the limited sample availability for this cohort, we chose to restrict our assessment of plasma cytokines to the least redundant IL-1 family members (i.e. IL-1β, IL-18 and IL-33). We expect that subjects with higher levels of IL-1β in this cohort are likely to have higher IL-1a and lower IL-1Ra. We hope to validate these findings in future mechanistic studies.

**Reviewer comment:** The fact that Itaconate is not significantly different following 12 months of treatment should be discussed more thoroughly as it might suggest alternative mechanisms to dampen inflammation.

**Author response:** We agree with the reviewer comment that persistent decreases in plasma concentrations of itaconate after treatment in the MDR-TB group suggests the proinflammatory immunometabolic network identified in the MDR-TB group is reversed by an alternative mechanism. We have discussed this more thoroughly in the revised manuscript (page 19, line 327-330).

**Reviewer comment:** Given IL1b is a key part to the story, are the authors able to examine whether signal 1 (Pro-IL1b) and/or signal 2 (inflammasome activation) are different based on their samples in hand? This may help drive at some mechanism given the constraints of these samples. If not maybe discuss these possibilities and what could drive each (ie more Mtb more TLR activation/Signal 1 or more cell damage so more inflammasome activation).

**Author response:** Our group has extensive expertise at detecting inflammasome complex proteins, caspase-1 and pro-IL1β using cellular assays like flow cytometry, imaging, and western blotting (PMID: 25311115). However, we were unable to conduct these experiments due to limited of availability of peripheral blood mononuclear cells. We agree with the reviewer that understanding the alterations in these molecules is important to decipher the mechanisms regulating the IL-1β production cascade and hope to undertake these experiments in follow-up studies. Based on the available literature, the observed metabolic remodeling could drive IL-1β secretion in multiple ways. Accumulation of succinate is known drive transcription of *Il1b* (PMID: 23535595) and would therefore be expected to result in greater production of pro-IL1β, while loss of itaconate-mediated inflammasome inhibition would be expected to increase inflammasome activation (PMID: 32791101). We have highlighted these different mechanisms in the revised manuscript (page 16, line 261 and page 18, line 319).
